# The novel curcumin analog FLLL32 decreases STAT3 DNA binding activity and expression, and induces apoptosis in osteosarcoma cell lines

**DOI:** 10.1186/1471-2407-11-112

**Published:** 2011-03-28

**Authors:** Stacey L Fossey, Misty D Bear, Jiayuh Lin, Chenglong Li, Eric B Schwartz, Pui-Kai Li, James R Fuchs, Joelle Fenger, William C Kisseberth, Cheryl A London

**Affiliations:** 1 Department of Veterinary Biosciences, The Ohio State University, Columbus, OH 43210, USA; 2 Department of Pediatrics, College of Medicine, The Ohio State University, Columbus, OH 43205, USA; 3 Comprehensive Cancer Center, The Ohio State University, Columbus, OH 43210, USA; 4 Division of Medicinal Chemistry and Pharmacognosy, College of Pharmacy, The Ohio State University, Columbus, OH 43210, USA; 5 Department of Veterinary Clinical Sciences, The Ohio State University, Columbus, OH 43210, USA

## Abstract

**Background:**

Curcumin is a naturally occurring phenolic compound shown to have a wide variety of antitumor activities; however, it does not attain sufficient blood levels to do so when ingested. Using structure-based design, a novel compound, FLLL32, was generated from curcumin. FLLL32 possesses superior biochemical properties and more specifically targets STAT3, a transcription factor important in tumor cell survival, proliferation, metastasis, and chemotherapy resistance. In our previous work, we found that several canine and human osteosarcoma (OSA) cell lines, but not normal osteoblasts, exhibit constitutive phosphorylation of STAT3. Compared to curcumin, we hypothesized that FLLL32 would be more efficient at inhibiting STAT3 function in OSA cells and that this would result in enhanced downregulation of STAT3 transcriptional targets and subsequent death of OSA cells.

**Methods:**

Human and canine OSA cells were treated with vehicle, curcumin, or FLLL32 and the effects on proliferation (CyQUANT^®^), apoptosis (SensoLyte^® ^Homogeneous AMC Caspase- 3/7 Assay kit, western blotting), STAT3 DNA binding (EMSA), and vascular endothelial growth factor (VEGF), survivin, and matrix metalloproteinase-2 (MMP2) expression (RT-PCR, western blotting) were measured. STAT3 expression was measured by RT-PCR, qRT- PCR, and western blotting.

**Results:**

Our data showed that FLLL32 decreased STAT3 DNA binding by EMSA. FLLL32 promoted loss of cell proliferation at lower concentrations than curcumin leading to caspase-3- dependent apoptosis, as evidenced by PARP cleavage and increased caspase 3/7 activity; this could be inhibited by treatment with the pan-caspase inhibitor Z-VAD-FMK. Treatment of OSA cells with FLLL32 decreased expression of survivin, VEGF, and MMP2 at both mRNA and protein levels with concurrent decreases in phosphorylated and total STAT3; this loss of total STAT3 occurred, in part, via the ubiquitin-proteasome pathway.

**Conclusions:**

These data demonstrate that the novel curcumin analog FLLL32 has biologic activity against OSA cell lines through inhibition of STAT3 function and expression. Future work with FLLL32 will define the therapeutic potential of this compound *in vivo*.

## Background

Osteosarcoma (OSA) is the most common form of malignant bone cancer in humans and dogs [1,2]. Multidrug chemotherapy and aggressive surgical techniques have improved survival; however, the prognosis for human patients with metastatic disease remains extremely poor with survival rates of 10-20% [3]. The disease in dogs occurs approximately 10 times more frequently than in people and treatment with surgery and adjuvant chemotherapy results in long-term survival rates of only 10-15% [4]. Both clinical and molecular evidence suggest that human and canine OSA share several key features including early metastasis, chemotherapy resistance, altered expression of several proteins (e.g., ezrin, Met, PTEN), and p53 mutation, among others [4-10]. Given these similarities, canine OSA serves as a relevant model in which to evaluate the potential clinical utility of novel therapeutic targets for this disease.

The transcription factor STAT3 has been implicated as a key player in several features of malignant neoplasia including tumor cell survival, metastasis, and resistance to chemotherapy [11-13]. Our data and the work of others support the notion that STAT3 may be a relevant target for therapy in both human and canine OSA. In previous work, we demonstrated that human and canine OSA cell lines and tumors from canine patients exhibited constitutive activation of STAT3 [14]. Loss of this expression after transfection with small interfering RNA targeting STAT3 or by reducing STAT3 DNA binding using LLL3 (a small molecule inhibitor) abrogated expression of STAT3 transcriptional targets and enhanced apoptosis [14]. Increased levels of phosphorylated STAT3 have been identified in a subset of human OSA tissue samples and cell lines supportive of the role of this transcription factor in OSA [15]. Suppression of this activated STAT3 with a dominant negative STAT3 led to decreased growth in these cell lines [15]. Studies by Wang *et al. *showed that inhibition of STAT3 expression in OSA cells by siRNA decreased proliferation and enhanced apoptosis of these cells [11]. Treatment of multidrug resistant OSA cell lines with a synthetic oleanane triterpenoid, C-28 methyl ester of 2-cyano-3,12-dioxoolen-1,9-dien-28-oic acid (CDDO-Me) downregulated STAT3 phosphorylation and nuclear translocation, subsequently inducing apoptosis [16]. Indeed, overexpression of phosphorylated STAT3 was associated with a poor prognosis in patients with OSA [17] and high levels of STAT3 protein were associated with metastasis [11]. Given the apparent role of STAT3 in the biology of OSA, clinically relevant therapies aimed at downregulating its activity would likely be therapeutically useful.

Curcumin (diferuloylmethane) is a naturally occurring compound found in the plant *Curcuma longa *that has numerous medicinal properties including anti-inflammatory and antitumor effects [18-20]. Curcumin has been investigated extensively as a potential therapeutic agent for the treatment of many different cancers, such as colorectal carcinoma [21,22], head and neck squamous cell carcinoma [23], pancreatic cancer [24], and OSA [25,26]. Curcumin is known to target multiple biochemical pathways, such as those mediated by Wnt/β-catenin [26], NF-κB [20], growth factor receptors like EGFR and HER2 [27], and JAK/STAT [28] enhancing its effect on cancer cells. Indeed, studies indicated that curcumin targets cellular transformation, invasion, angiogenesis, and metastasis [27,29-32]. Recent work demonstrated that curcumin induced cell cycle arrest and apoptosis, and inhibited migration in human OSA cell lines [19,33]. However, curcumin is not stable under physiologic conditions and is not readily absorbed after ingestion [34]. Multiple modifications to the structure of curcumin have been investigated in an attempt to improve potency and biochemical properties [18,35-37].

Recent work on improving both the target specificity and stability of curcumin by the College of Pharmacy at The Ohio State University produced the novel small molecule STAT3 inhibitor, FLLL32. As a diketone analog of curcumin, FLLL32 is more selective in its targeting than the parent compound due to the replacement of two hydrogen atoms on the central carbon of curcumin with a spiro-cyclohexyl ring [38]. Improved interaction of FLLL32 with the Src homology-2 (SH2) domain of STAT3, a region instrumental in its dimerization and nuclear translocation, as well as greater stability, was predicted with these modifications as compared to curcumin [38,39]. In subsequent work, FLLL32 was shown to promote apoptosis in multiple human cancer cell lines, inducing downregulation of STAT3 phosphorylation and DNA binding [38-40]. In human hepatocellular cancer cells, FLLL32 inhibited IL-6-induced STAT3 phosphorylation [41]. FLLL32 was found to be more potent than some existing STAT3 inhibitors, including Stattic, S3I-201, and curcumin in colorectal, glioblastoma, multiple myeloma, rhabdomyosarcoma, and liver cancer cell lines [39,42]. Together, these data demonstrate that FLLL32 exhibits improved efficacy at abrogating STAT3 functional activity and its effects in enhancing tumor cell survival in many cancer cell lines as compared to curcumin and other STAT3 inhibitors. Therefore, the purpose of this study was to explore the biologic activity of FLLL32 against canine and human OSA cell lines *in vitro*, delineate the mechanism of action of FLLL32, and compare the efficacy of FLLL32 to curcumin.

## Methods

### Cell Lines and Reagents

Canine OSA cell lines, OSA 8 and 16 were provided by Dr. Jaime Modiano (University of Minnesota, Minneapolis, MN) [43,44]. The canine D17 OSA cell line and human OSA cell lines U2OS and SJSA were purchased from American Type Cell Culture Collection (ATCC, Manassas, VA). Cell line authentication of human OSA cell lines SJSA and U2OS was recently completed by The Ohio State University Comprehensive Cancer Center Molecular Cytogenetics Shared Resource by comparing the ATCC karyotype features with that of our cell lines. The canine lines and human line SJSA were maintained in RPMI-1640 supplemented with 10% fetal bovine serum, non-essential amino acids, sodium pyruvate, penicillin, streptomycin, L-glutamine, and HEPES (4-(2-hydroxyethyl)-1-piperazineethanesulfonic acid) at 35°C, supplemented with 5% CO_2_. The remaining human cell line U2OS was cultured in McCoy's medium with 10% FBS and the same supplements as listed for the canine lines. FLLL32 was synthesized and purified as described previously [38]. Curcumin, the proteasome inhibitor MG132, and the pan-caspase inhibitor, Z-VAD-FMK, were purchased from EMD Chemicals (Gibbstown, NJ).

### Cell proliferation

OSA cells (2 × 10^3^) were seeded in 96-well plates overnight and incubated with DMSO, 10 μM curcumin, or increasing concentrations of FLLL32 (0.25, 0.75, 2.5, or 7.5 μM) for 72 hours. The volume of DMSO added to the vehicle treated wells was the same as that added to the drug treated wells. Each drug concentration was performed in four replicate wells. The media was removed, the wells were washed with PBS, and the plates were frozen at -80°C overnight before processing with the CyQUANT^® ^Cell Proliferation Assay Kit (Molecular Probes, Eugene, OR) as described previously [14]. Cell proliferation was calculated as a percentage of the DMSO- treated control wells with IC_50 _values derived after plotting proliferation values on a logarithmic curve.

### Detection of Apoptosis/Caspase 3/7 Activity

OSA cells (1.1 × 10^4^) were seeded in 96-well plates overnight and incubated with media, DMSO, 10 μM curcumin, or FLLL32 (2.5 or 7.5 μM) for 24 hours. Wells with media only were included as controls. Levels of caspase- 3/7 activity were determined using the SensoLyte^® ^Homogeneous AMC Caspase- 3/7 Assay kit (Anaspec Inc, San Jose, CA) as described previously [14]. To determine the effect of caspase activation on the loss of STAT3 protein, 1.1 × 10^4 ^OSA cells were pretreated for either 2 or 24 hours with 80 μM Z-VAD-FMK. Cells were then treated for 18 hours with media, DMSO, 80 μM Z-VAD-FMK, 10 μM FLLL32, or 10 μM FLLL32 and 80 μM Z-VAD-FMK. Caspase activation was measured as described previously [14].

### EMSA

To confirm that FLLL32 impaired STAT3 DNA binding, we used the Pierce LightShift Chemiluminescent EMSA kit (Thermo Fisher Scientific Inc, Rockford, IL) that employs a chemiluminescent detection system to detect protein:DNA interactions as described previously [14]. Briefly, nuclear protein from human (0.8 μg/well) and canine (5 μg/well) OSA cell lines treated for four hours with media, DMSO, 10 μM curcumin, or 10 μM FLLL32 was collected using the NucBuster™ Protein Extraction kit (EMD Chemicals Inc, Gibbstown, NJ). Protein from cell lysates was collected from each group concurrently and processed for western blotting as described previously to confirm levels of STAT3 total protein and β-actin.

### RT-PCR and qRT-PCR

RNA was extracted from canine and human OSA cells following 12-24 hours treatment with DMSO, curcumin, or FLLL32 using TRIzol reagent (Invitrogen, Carlsbad, CA) according to the manufacturer's instructions. To generate cDNA, 2 μg of total RNA and the M-MLV reverse transcriptase kit (Invitrogen, Carlsbad, CA) were used according to the manufacturer's instructions. Next, 1/20 of the resultant cDNA was used for each PCR reaction in a total volume of 25 μl. Primers designed and utilized for canine STAT3 are listed in Table 1; the annealing temperature for this reaction was 55°C. Primers designed and utilized for canine STAT3 transcriptional targets VEGF and MMP2 and GAPDH and human VEGF and GAPDH were published previously with annealing temperatures [14]. Primers designed and utilized for human STAT3 and MMP2 are listed in Table 1. An annealing temperature of 60ºC was used for PCR reactions with human primers for STAT3 and MMP2. Primers were designed to span at least one intron to identify and eliminate any potential genomic DNA contamination. All PCR products were run on a 2% agarose gel with ethidium bromide and visualized using the Alpha Imager system (Alpha Innotech Corp, San Leandro, CA).

**Table 1:** Primers for human and canine reverse transcriptase polymerase chain reactions

Primers	Primer Sequences
Canine STAT3F	5'- GGC CCA ATG GAA TCA GCT ACA G -3'
Canine STAT3R	5'- GAA GGA ACT GCT TGA TTC TTC G -3'
Human STAT3F	5'- GGC CCA ATG GAA TCA GCT ACA G -3'
Human STAT3R	5'- GAA GAA ACT GCT TGA TTC TTC G -3'
Human MMP2F	5'- GAT GGC ACC CAT TTA CAC CTA C -3'
Human MMP2R	5'- GTC CTT GAA GAA GAA GAT CTC -3'

To quantitatively measure the effects of treatment on STAT3 expression, canine OSA cells (OSA8) were treated with curcumin or FLLL32 for 4 or 24 hours, and RNA was extracted using TRIzol reagent (Invitrogen, Carlsbad, CA) according to the manufacturer's instructions. cDNA was made from 1 μg total RNA using the Superscript III kit (Invitrogen). Real-time quantitative PCR was performed using the Applied Biosystem's StepOne Plus Real-Time PCR System. STAT3 and 18S mRNA were detected using Fast SYBR green PCR master mix (Applied Biosystems) according to the manufacturer's protocol and primer sets are detailed in Table 2. All reactions were performed in triplicate and included no-template controls for each gene. Relative expression was calculated using the comparative threshold cycle method [45]. Experiments were repeated 3 times using samples in triplicate.

**Table 2:** Primers for STAT3 quantitative reverse transcriptase polymerase chain reaction

Primers	Primer Sequences
Canine STAT3F	5'-GGC CCA ATG GAA TCA GCT ACA G-3'
Canine STAT3R	5'-GAA GGA ACT GCT TGA TTC TTC G-3'
18S V2F	5'-AAA TCC TTT AAC GAG GAT CCA TT-3'
18S V2R	5'-AAT ATA CGC TAT TGG AGC TGG A-3'

### Western Blotting

Protein lysates were prepared and quantified, separated by SDS-PAGE, and Western blotting was performed using previously described methods [14] on 2 × 10^6 ^OSA cells after treatment with either curcumin, FLLL32, or DMSO for 24 hours. The membranes were then incubated overnight with anti-p-STAT3 (Tyr705, Cell Signaling Technology, Danvers, MA), anti-p-ERK1/2 (Thr202/Tyr204, Cell Signaling Technology, Danvers, MA), anti-PARP (BD Biosciences, San Jose, CA), anti-VEGF (Calbiochem, Gibbstown, NJ), anti-ubiquitin (Cell Signaling Technology, Danvers, MA), or anti-survivin antibody (Novus Biologicals, Littleton, CO). The membranes were incubated with appropriate horseradish peroxidase linked secondary antibodies, washed, and exposed to substrate (SuperSignal West Dura Extended Duration Substrate, Pierce, Rockford, IL). Blots were stripped, washed, and reprobed for β-actin (Santa Cruz Biotechnology, Santa Cruz, CA), total STAT3 (Cell Signaling Technology, Danvers, MA) or total ERK1/2 (Cell Signaling Technology, Danvers, MA).

### Immunoprecipitation

OSA cells (7 × 10^6^) were serum starved for two hours then treated with DMSO, 10 μM curcumin, 10 μM FLLL32, or 10 μM MG132 for 4 hours. The volume of DMSO added to the vehicle treated wells was the same as that delivered to the drug treated wells. Cells were collected and lysate prepared as described previously [14]. STAT3 antibody (Cell Signaling Technology, Danvers, MA) was added to lysates that had been precleared with Protein A- Agarose beads (Roche Diagnostics, Indianapolis, IN) and allowed to incubate overnight at 4°C. Protein A- Agarose beads were added to the protein lysate and antibody and incubated 1 hour at 4°C then washed three times in cold lysis buffer. This was spun down and supernatant separated by SDS-PAGE and transferred to a PVDF membrane (Thermo Scientific, Rockford, IL). Western blotting using an anti-ubiquitin antibody (Cell Signaling Technology, Danvers, MA) was performed after addition of the appropriate secondary antibody. The membrane was stripped and reprobed for total STAT3 (Cell Signaling Technology, Danvers, MA) or β-actin (Santa Cruz Biotechnology, Santa Cruz, CA). Images were scanned and analyzed using Image J (Rasband, W. S., Image J, U. S. National Institutes of Health, Bethesda, Maryland, USA, http://rsb.info.nih.gov/ij/, 1997-2009).

### Proteasome Inhibition Assay

OSA cells (6 × 10^6^) were serum starved for 2 hours then treated with DMSO, 10 μM curcumin, 10 μM FLLL32, or 10 μM MG132 for 4 hours. After treatment, cells were collected, washed with cold PBS, and lysed. Cell lysis buffer contained 50 mM HEPES (pH 7.5), 5 mM ethylenediaminetetraacetic acid (EDTA), 150 mM sodium chloride, and 1% Triton X-100. Proteasome enzyme was extracted and prepared for use in the 20S Proteasome Activity Assay Kit (Millipore, Billerica, MA). The 20S proteasome activity was measured in a 96-well plate. The assay is based on detection of the fluorophore 7-amino-4-methylcoumarin (AMC) after cleavage from labeled substrate LLVY-AMC. Samples were incubated for 1 hour at 37°C prior to detection of free AMC fluorescence using a 380/460 nm filter set in a SpectraMax microplate reader (Molecular Devices Corp., Sunnyvale, CA).

### Statistical Methods

Statistical analysis of the CyQUANT^® ^proliferation assays, caspase 3/7 activity, and real time PCR data was performed using the Student's t-test. *P *values of < 0.05 were considered statistically significant.

## Results

### Treatment with curcumin or FLLL32 decreased proliferation of OSA cell lines

**Figure 1:**
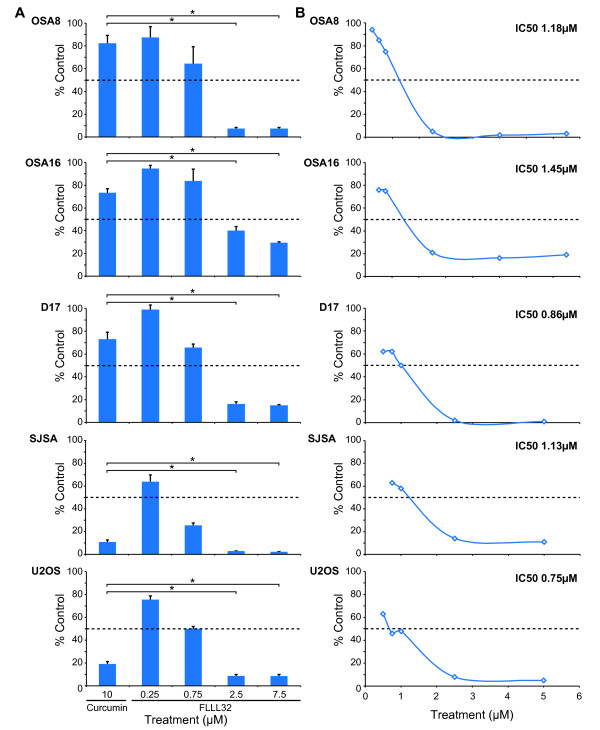
**Treatment with curcumin or FLLL32 decreased the proliferation of OSA cell lines**. **A) **Canine (OSA8, OSA16, and D17) or human OSA cell lines (SJSA and U2OS) were treated with vehicle, curcumin, or FLLL32 for 72 hours. Proliferation was analyzed using the CyQUANT^® ^cell proliferation assay kit. Proliferation values are listed as a percentage of DMSO control. Experiments were performed in quadruplicate and repeated two times. Statistical analysis of cell proliferation was performed using the Student's t-test. *P *values of < 0.05 were considered statistically significant.*p < 0.05 **B) **Canine and human OSA cell lines were treated with DMSO or FLLL32 for 72 hours and analyzed with CyQUANT^® ^to determine proliferation as a percentage of DMSO control. IC_50_s for FLLL32 were calculated for each cell line using a log curve. Experiments were performed in quadruplicate and repeated two times.

Canine (OSA8, 16, and D17) and human (SJSA and U2OS) OSA cell lines were treated with 10 μM curcumin or increasing concentrations of FLLL32 for 72 hours and proliferation was measured. Figure 1A shows that both canine and human OSA cell lines exhibited significant decreases in proliferation after treatment with FLLL32, particularly at concentrations above 0.75 μM. Interestingly, while the human cell lines were sensitive to curcumin treatment, the canine lines appeared to be somewhat resistant. However, FLLL32 induced a statistically significant greater effect on proliferation of all OSA cell lines at lower concentrations (2.5 μM and 7.5 μM) when compared to that induced by curcumin at 10 μM. As depicted in Figure 1B, the IC_50 _for FLLL32 ranged from 0.75-1.45 μM for the OSA cell lines as extrapolated from logarithmic curves. These data demonstrate that FLLL32 is more potent than curcumin, with FLLL32 inhibiting cell proliferation at lower concentrations than curcumin both in canine and human OSA cell lines.

### FLLL32 induced activation of caspase 3/7, PARP cleavage, and apoptosis of OSA cell lines

**Figure 2:**
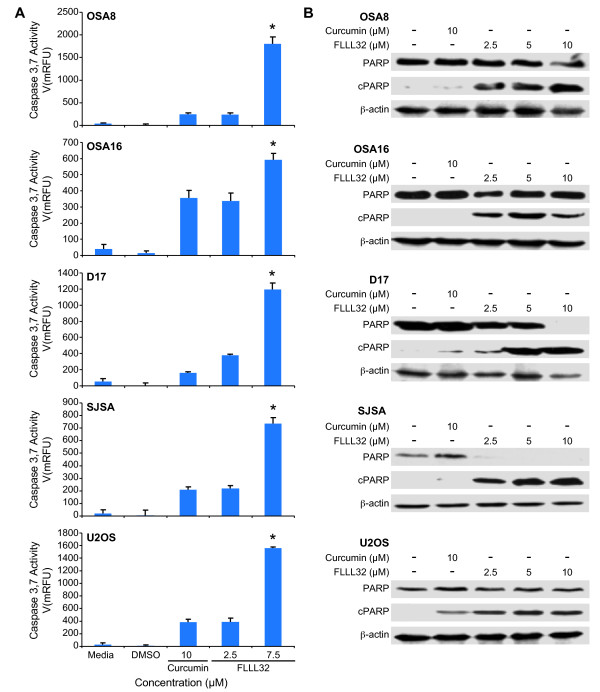
**FLLL32 induced activation of caspase 3/7, PARP cleavage, and apoptosis of OSA cell lines**. **A) **Canine (OSA8, OSA16, and D17) and human (SJSA and U2OS) OSA cell lines were treated with media, DMSO, curcumin, or FLLL32 for 24 hours. Apoptosis was assessed by measuring active caspase-3/7 using the SensoLyte^® ^Homogeneous AMC Caspase-3/7 Assay kit. Experiments were performed in triplicate and repeated two times. Statistical analysis of the caspase 3/7 activity was performed using the Student's t-test. *P *values of < 0.05 were considered statistically significant.*p < 0.05 **B) **Canine (OSA8, OSA16, and D17) or human (SJSA and U2OS) OSA cell lines were treated with DMSO, curcumin, or FLLL32 for 24 hours prior to collection. Protein lysates were generated and separated by SDS-PAGE and western blotting for PARP and β-actin was performed. Experiments were repeated two times.

Previous work in our laboratory demonstrated that siRNA mediated downregulation of STAT3 expression in human and canine OSA cell lines induced apoptosis [14]. To evaluate the effects of FLLL32 on OSA cells, canine and human OSA cell lines were cultured with curcumin or increasing concentrations of FLLL32 for 24 hours and apoptosis was measured. Significant increases in caspase 3/7 activity occurred at 7.5 μM of FLLL32 compared to curcumin at 10 μM (Figure 2A). Additionally, we examined the status of poly (ADP-ribose) polymerase (PARP), a nuclear enzyme important for chromosomal structure and genomic stability [46]. PARP cleavage occurs following caspase-3 activation during the process of apoptosis [47]. A dose-dependent increase in PARP cleavage (cPARP) in both canine and human OSA cell lines also occurred after 24 hours of treatment with FLLL32 (Figure 2B). In contrast, there was minimal to no PARP cleavage induced by treatment with 10 μM curcumin (Figure 2B).

### FLLL32 decreased STAT3 DNA binding in OSA cell lines

**Figure 3:**
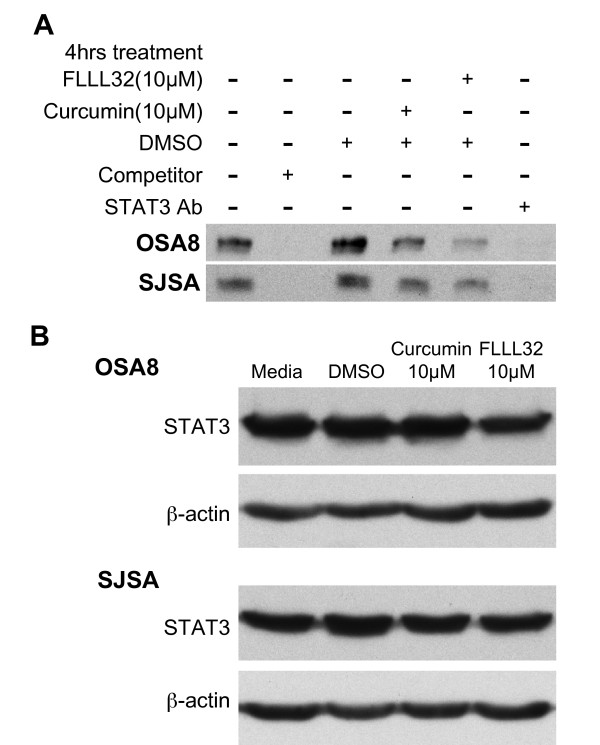
**FLLL32 decreased STAT3 DNA binding in OSA cell lines**. **A) **Canine OSA cell line OSA8 and human OSA cell line SJSA were incubated with media, DMSO, 10 μM curcumin, or 10 μM FLLL32 for 4 hours. Cells were harvested and nuclear protein isolated. Nuclear protein was added to binding reactions with labeled species specific DNA probes for the STAT3 recognition sequences located in the promoter for survivin in the presence or absence of unlabelled competitor probe. Additionally, anti-STAT3 antibody was added to nuclear protein from cells treated with media alone to demonstrate specificity of the binding reaction. Reactions were separated on an acrylamide gel, transferred to a nylon membrane, and the DNA was crosslinked. The membranes were processed using the LightShift Chemiluminescent EMSA kit (Thermo Fisher Scientific Inc, Rockford, IL). Experiments were repeated two times. **B) **Canine OSA cell line OSA8 and human OSA cell line SJSA were incubated with media, DMSO, 10 μM curcumin, or 10 μM FLLL32 for 4 hours concurrently with cells treated for EMSA above. Cells were harvested and total protein isolated and quantified. Protein was separated by SDS-PAGE. Western blotting was performed for STAT3 and β-actin. Experiments were repeated two times.

The curcumin analog FLLL32 acts in part through direct inhibition of STAT3 DNA binding by interacting with its SH2 domain, which is critical for dimerization [38]. We observed that both canine (OSA8) and human (SJSA) OSA cells exhibited decreased STAT3 DNA binding after only 4 hours of treatment with curcumin or FLLL32 (Figure 3A). To determine if the decrease in DNA binding was due to loss of STAT3 total protein, we harvested protein from cells concurrently treated for 4 hours and observed no significant decrease in STAT3 protein compared to media or DMSO- treated cells (Figure 3B).

### Downregulation of STAT3 via FLLL32 treatment decreased expression of VEGF, MMP2, and survivin

**Figure 4:**
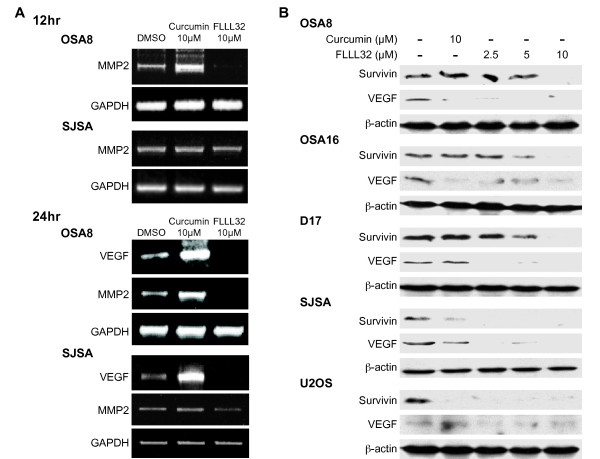
**Downregulation of STAT3 via FLLL32 treatment decreased expression of VEGF, MMP2, and survivin**. **A) **Canine (OSA8) or human (SJSA) OSA cell lines were treated with DMSO, 10 μM curcumin, or 10 μM FLLL32 for 12 or 24 hours. RNA was collected and RT-PCR was performed for VEGF, MMP2, and GAPDH. Experiments were repeated two times. **B) **Canine (OSA8, OSA16, and D17) or human (SJSA and U2OS) OSA cell lines were treated with DMSO, curcumin, or FLLL32 for 24 hours prior to collection. Protein lysates were generated and separated by SDS-PAGE and western blotting for survivin, VEGF, and β-actin was performed. Experiments were repeated two times.

Given the role of survivin, VEGF, and MMP2 in tumor cell survival, angiogenesis, and metastasis, we determined if downregulation of STAT3 DNA binding correlated with loss of expression of these STAT3 transcriptional targets in OSA cell lines. Canine (OSA8) and human (SJSA) OSA cells were treated for 12 or 24 hours with DMSO, 10 μM curcumin, or 10 μM FLLL32. Loss of MMP2 mRNA expression occurred in OSA8 at both 12 and 24 hours after treatment with 10 μM FLLL32; however, loss of MMP2 mRNA in the SJSA line was not noted until 24 hours of FLLL32 exposure (Figure 4A). Treatment with 10 μM FLLL32 resulted in loss of VEGF mRNA expression in both cell lines after 24 hours of drug treatment (Figure 4A). Additionally, downregulation of VEGF protein expression was similarly observed following 24 hours of FLLL32 exposure at 10 μM and was also noted at lower concentrations (2.5 and 5 μM) of drug (Figure 4B). Interestingly, VEGF mRNA levels appeared to be increased in the OSA8 and SJSA lines after 24 hours of exposure to 10 μM curcumin, although this did not correlate with the observed changes in VEGF protein in which VEGF was unchanged (SJSA) or downregulated (OSA8) after curcumin treatment. Decreases in survivin expression occurred at 5 and 10 μM FLLL32 in the canine OSA lines and at 2.5 μM FLLL32 and higher in the human OSA lines (Figure 4B). Curcumin downregulated survivin expression in the human but not canine OSA lines, supporting the notion that, as with the previously discussed proliferation data, the human cells are much more sensitive to the effects of curcumin.

### Treatment with FLLL32 decreased pSTAT3 and total STAT3 expression in canine and human OSA

**Figure 5:**
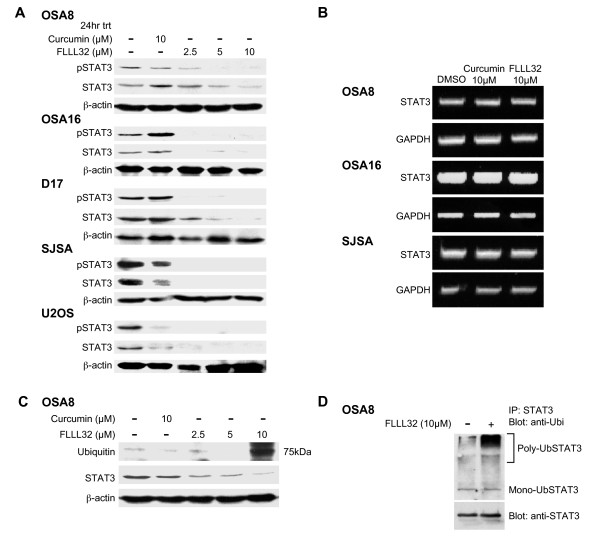
**Treatment with FLLL32 decreased pSTAT3 and total STAT3 expression in canine and human OSA cell lines**. **A) **Canine (OSA8, OSA16, and D17) or human (SJSA and U2OS) OSA cell lines were treated with DMSO, curcumin, or FLLL32 for 24 hours prior to collection. Protein lysates were generated and separated by SDS-PAGE and western blotting for pSTAT3 (Y705), total STAT3, and β-actin was performed. Experiments were repeated two times. **B) **Canine (OSA8 and 16) or human (SJSA) OSA cell lines were treated with DMSO, 10 μM curcumin, or 10 μM FLLL32 for 24 hours. RNA was collected and RT-PCR was performed for STAT3 and GAPDH. Experiments were repeated two times. **C) **Canine OSA cell line OSA8 was treated with DMSO, curcumin, or FLLL32 for 24 hours prior to collection. Protein lysates were generated and separated by SDS-PAGE and Western blotting for ubiquitin, STAT3, and β-actin was performed. Experiments were repeated two times. **D) **OSA8 was treated with DMSO or FLLL32 for 4 hours prior to collection. Protein lysates were generated and STAT3 was immunoprecipitated. Protein was separated by SDS-PAGE and western blotting for ubiquitin and STAT3 was performed. Experiments were repeated two times.

Human and canine OSA cells were treated with 10 μM curcumin or increasing concentrations of FLLL32 for 24 hours to determine their effect on STAT3 phosphorylation. There was a dose dependent decrease in STAT3 tyrosine 705 phosphorylation as demonstrated by Western blotting with downregulation occurring at 2.5 μM FLLL32 (Figure 5A). Additionally, decreases in total STAT3 occurred after FLLL32 treatment in all cell lines treated (Figure 5A). To determine the mechanism for loss of total STAT3 protein, we treated canine and human OSA cell lines with FLLL32 for 24 hours and performed RT-PCR to determine whether this was due to loss of *stat3 *gene expression as STAT3 is known to regulate its own expression through an autoregulatory loop [48]. Using standard RT-PCR, there was no downregulation of STAT3 mRNA expression after 24 hours with treatment with curcumin or FLLL32 (Figure 5B). When OSA8 cells were treated with FLLL32 and STAT3 expression was evaluated using quantitative real time PCR, a small decrease in STAT3 mRNA expression was present at 24 hours, but this was not statistically significant (Additional File 1) and therefore would be unlikely to account for the protein loss observed by western blotting. Lastly, the loss of STAT3 was not due to global loss of proteins secondary to cell death as there were no differences in the levels of pERK1/2 and total ERK 1/2 in OSA cell lines treated with drug for 24 hrs (Additional File 2).

### STAT3 downregulation after FLLL32 treatment occurred through the ubiquitin/proteasome pathway

**Figure 6:**
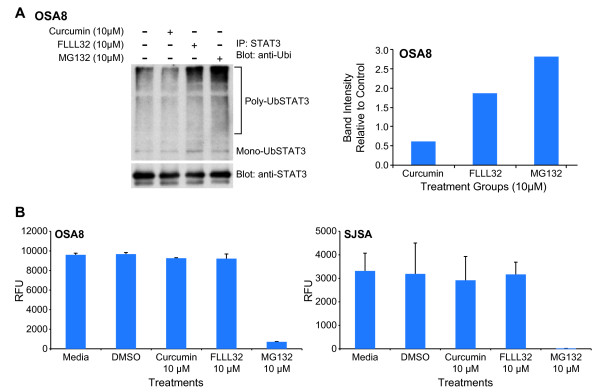
**Loss of STAT3 occurred in part through the ubiquitin-proteasome pathway however curcumin and FLLL32 did not inhibit the 20S proteasome in OSA cell lines**. **A) **OSA8 was treated with DMSO, curcumin, FLLL32, or the proteasome inhibitor MG132 for 4 hours prior to collection. Protein lysates were generated and STAT3 was immunoprecipitated. Protein was separated by SDS-PAGE and western blotting for ubiquitin and STAT3 was performed. Experiments were repeated two times. Densitometry analysis was performed using Image J (Rasband, W. S., Image J, U. S. National Institutes of Health, Bethesda, Maryland, USA, http://rsb.info.nih.gov/ij/, 1997-2009). **B**) Canine (OSA8) or human (SJSA) OSA cells were serum starved for 2 hours then treated with DMSO, 10 μM curcumin, 10 μM FLLL32, or 10 μM MG132 for 4 hours. Cells were collected, washed with cold PBS, and prepared for use in the 20S Proteasome Activity Assay Kit (Millipore, Billerica, MA). Experiments were repeated two times.

STAT family proteins are known to be regulated by ubiquitin-mediated degradation [49,50]. To determine if this mechanism was responsible for the loss of total STAT3 following FLLL32 treatment, the OSA8 cell line was treated with curcumin or FLLL32 for 24 hours and Western blotting for ubiquitin was performed on lysates. An intense band emerged at 75 kDa in FLLL32 treated cells corresponding to the size of STAT3 (Figure 5C). We next immunoprecipitated STAT3 and performed Western blotting for ubiquitin. A band was present at 75 kDa in addition to a smear directly above the band in the group treated with 10 μM FLLL32 for 4 hours (Figure 5D). This was interpreted to be mono-ubiquitinylated STAT3 at 75 kDa and poly-ubiquitinylated STAT3 protein at the large molecular weight sizes. Indeed, after treating OSA8 cells with curcumin, FLLL32, or the proteasome inhibitor MG132, there was almost a four-fold increase in poly-ubiquitinylated STAT3 in cells treated with FLLL32 as compared to those treated with curcumin (Figure 6A). Immunoblotting for β-actin was performed to confirm the specificity of the immunoprecipitation experiment; none was detected (data not shown). Although it has been reported that curcumin has proteasome inhibition properties [51], treatment with curcumin or FLLL32 did not lead to alteration in the activity of the 20S proteasome when compared with MG132 at the same concentration (Figure 6B).

### Inhibition of caspase activation did not affect loss of STAT3 following FLLL32 treatment

**Figure 7:**
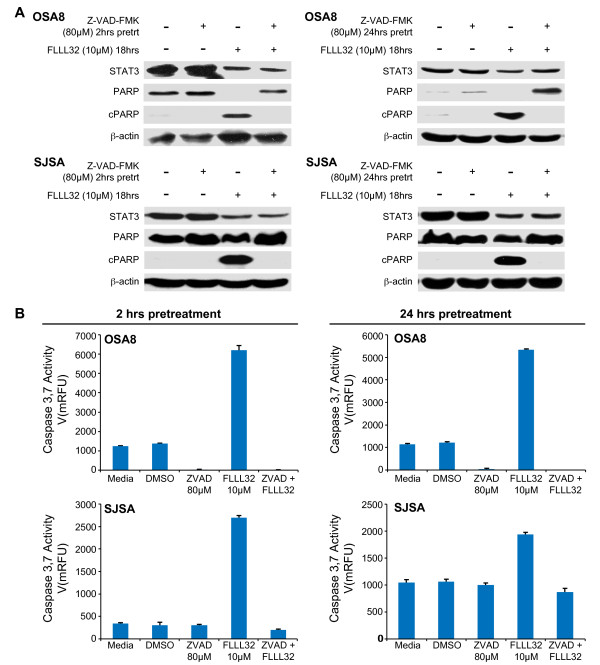
**Inhibition of caspase activation did not affect loss of STAT3 following FLLL32 treatment**. **A) **Canine (OSA8) and human (SJSA) OSA cell lines were pretreated with the pan-caspase inhibitor 80 μM Z-VAD-FMK or DMSO for 2 or 24 hours then treated with DMSO or 10 μM FLLL32 for 18 hours. Protein lysates were generated and separated by SDS-PAGE and western blotting for STAT3, PARP, and β-actin was performed. Experiments were repeated two times. **B**) Canine (OSA8) or human (SJSA) OSA cell lines were pretreated with the pan-caspase inhibitor 80 μM Z-VAD-FMK, DMSO, or media for 2 or 24 hours then treated with media, DMSO, or 10 μM FLLL32 for 18 hours. Caspase-3/7 activity was measured using the SensoLyte^® ^Homogeneous AMC Caspase-3/7 Assay kit. Experiments were performed in triplicate and repeated two times.

Activated caspases 2, 4, 5, and 10 are known to be capable of cleaving STAT3 [52]. To investigate whether loss of STAT3 after treatment with FLLL32 was due to cleavage by activated caspases, we pretreated the OSA8 and SJSA cell lines with a pan-caspase inhibitor Z-VAD-FMK for 2 or 24 hours and then added FLLL32 or DMSO to the cells for an additional 18 hours. Western blotting of cell lysates demonstrated that inhibition of caspase activity by Z-VAD-FMK abrogated PARP cleavage but it did not significantly alter the amount of total STAT3 remaining after FLLL32 treatment compared with cells treated with FLLL32 and no Z-VAD-FMK (Figure 7A). Furthermore, Z-VAD-FMK pretreatment abrogated caspase 3/7 activation but this had no effect on the loss of STAT3 following FLLL32 treatment (Figure 7B). These data indicate that loss of STAT3 protein after FLLL32 exposure was not due to caspase- mediated cleavage.

## Discussion

Curcumin has a long history of use as a medicinal compound and is known to have multiple anti-inflammatory and anti-cancer properties; however, blood levels that can be achieved after oral administration are low, which limits its potential clinical value [20,27,34]. Curcumin also affects a broad range of cellular targets including STAT3 [53,54] in addition to a host of other signaling molecules such as Wnt/β-catenin [26], NF-κB [20], and HER2 [27], and the proteasome [51]. Given the number of targets affected by curcumin and its poor bioavailability, efforts have been directed at improving its chemical properties by complexing it with lipids/phospholipids [55,56] and developing more specific derivatives [35,57-59]. Interestingly, many of these analogues have demonstrated greater stability and more potent activity against several tumor cell lines, including those derived from breast, prostate, pancreas, and colon cancers when compared to curcumin [35,57-59]. Curcumin has been found to be well-tolerated in healthy individuals and OSA patients [18], most recently when given as a solid lipid particle formulation. However, peak plasma levels reached only 22.43 ng/mL (approximately 60 nM), well below concentrations known to have biologic effects against OSA cells *in vitro*.

During the development of novel curcumin analogs, our collaborators determined that one of these compounds, FLLL32, was particularly effective at suppressing the growth of pancreatic and breast cancer cells [38]. To produce FLLL32, the two hydrogen atoms on the central carbon of curcumin were replaced with a spiro-cyclohexyl ring. It was proposed that this alteration would confer greater stability and specificity for STAT3 than curcumin [38]. Recent work with FLLL32 showed that it induced apoptosis in human melanoma, multiple myeloma, glioblastoma, pancreatic, breast, and colorectal cancer cell lines and inhibited STAT3 phosphorylation and DNA binding [38-40]. The compound also exhibited higher potency at inhibiting proliferation and STAT3 DNA binding activity than curcumin and other JAK/STAT3 inhibitors in human rhabdomyosarcoma cells [42]. Indeed, FLLL32 has been shown to be more potent than other STAT3 inhibitors in promoting growth inhibition of multiple cancer cell lines, and the drug is improved in its specificity as demonstrated by kinase profile assays that revealed almost no activity against tyrosine kinases such as Lck, Syk, Lyn, Yes, and Abl-1 [39]. Given the superior specificity and efficacy of FLLL32 as compared to curcumin in a variety of cancer cell lines, the purpose of this study was to evaluate the biologic activity of this compound against OSA cell lines.

Previous studies have explored the activity of curcumin against OSA both *in vitro *and in human clinical trials [18,20,27]. OSA cell lines experienced cell cycle arrest, reduced proliferation, and underwent apoptosis following treatment with curcumin [19,33,60]. Prior work in our laboratory demonstrated that STAT3 is constitutively activated in OSA cell lines and that inhibition of STAT3 through STAT3 siRNAs or the small molecule STAT3 inhibitor LLL3 resulted in loss of proliferation and apoptosis [14]. Data presented in this study showed that FLLL32 inhibited proliferation of OSA cell lines and promoted apoptosis via caspase 3/7 activation at lower concentrations than curcumin. This is consistent with recent work demonstrating apoptosis via caspase activation in human multiple myeloma, glioblastoma, liver cancer, colorectal, and melanoma cell lines after FLLL32 exposure [39,40]. Cleavage of PARP, an indicator of caspase-3-mediated apoptosis, was also seen in many of these human cancer cell lines upon treatment with FLLL32 [39]. Interestingly, loss of messenger RNA and protein expression of survivin, an inhibitor of apoptosis, as well as decreased STAT3 DNA binding activity was observed in human rhabdomyosarcoma cells treated with FLLL32 [42]. The concurrent reduction in STAT3 transcriptional activity of targets such as survivin through decreased DNA binding and loss of STAT3 phosphorylation likely both played a role in the reduced survival of OSA tumor cells observed following exposure to FLLL32.

Recent work has shown that expression of high levels of STAT3 in human OSA tumor samples correlated to poor differentiation, metastasis, and lower rates of overall and relapse-free survival [11]. Overexpression of phosphorylated STAT3 in OSA has also been linked to poor prognosis [17]. STAT3 is known to enhance tumor cell invasion, metastasis, and angiogenesis through enhanced expression of VEGF and MMP2 [61]. Human patients with OSA whose tumors had higher VEGF expression as shown by immunohistochemistry had a significantly worse prognosis and had lung metastasis [62,63]. Previous work revealed that treatment of OSA cell lines with curcumin inhibited their migration [26]. Mouse xenograft models of pancreatic and colorectal cancer treated with curcumin exhibited suppression of tumor angiogenesis and tumor growth inhibition [20]. In more recent studies, FLLL32 inhibited vascularity and tumor growth in chicken embryo xenografts and reduced tumor volume in mouse xenografts of breast cancer [38]. Our data demonstrate that in the OSA cell lines we tested, VEGF mRNA and protein and MMP2 mRNA were expressed and treatment with 10 μM FLLL32 downregulated the expression of these STAT3 transcriptional targets following 24 hours of drug exposure. Interestingly, VEGF mRNA expression appeared to increase over baseline in both the OSA8 and SJSA lines after curcumin exposure, although this did not correlate with the findings obtained by Western blotting in which VEGF protein was absent in OSA8 cells and unchanged in SJSA cells. The mechanism for this observed discrepancy is not clear, although there are several possible explanations. Curcumin may somehow interfere with translation of VEGF mRNA, directly enhance degradation of VEGF protein, or alternatively, given its diversity of cellular targets, affect proteins other than STAT3 that in turn alters VEGF expression. Further investigation of these potential mechanisms is needed. Given the putative role of both VEGF and MMP2 in the process of tumor growth and metastasis and recent data demonstrating the ability of FLLL32 to abrogate breast cancer xenograft growth in mice, future work assessing the effects of FLLL32 in mouse models of OSA is warranted.

Treatment of OSA cell lines with FLLL32 promoted loss of both pSTAT3 and total STAT3. This loss of STAT3 correlated with the presence of mono- and polyubiquitinylated STAT3, indicating that proteasome-mediated degradation was likely responsible for the observed decrease in protein. Interestingly, curcumin has been shown to inhibit activities of the proteasome in certain cancer cells [51]; however we detected no evidence for this activity after treating the OSA cell lines with curcumin or FLLL32 at the doses and time points examined. Although modulation of STAT3 protein levels is known to occur in part through caspase cleavage [52] a pan-caspase inhibitor did not affect the observed loss of STAT3 after FLLL32 treatment. Additionally, we did not see a significant decrease in STAT3 mRNA 24 hours after FLLL32 treatment, indicating that loss of STAT3 mRNA could not be primarily responsible for the protein downregulation that occurs after FLLL32 exposure. These data support the assertion that in addition to blocking STAT3 function, FLLL32 acts to promote downregulation of STAT3 protein, thereby enhancing the functional consequences of this small molecule inhibitor.

## Conclusions

The novel small molecule STAT3 inhibitor FLLL32 downregulated proliferation and promoted apoptosis of OSA cells. FLLL32 inhibited STAT3 DNA binding and induced proteasome mediated degradation of STAT3 resulting in a subsequent loss of VEGF, MMP2, and survivin expression. These data support the notion that STAT3 is a relevant target for therapeutic intervention in OSA and that FLLL32 and similar analogs may have clinical utility for the treatment of OSA.

## Competing interests

The authors declare that they have no competing interests.

## Authors' contributions

SF designed and carried out molecular experiments on OSA cell lines and drafted the manuscript. MB participated in RT-PCR design and performance. JL, CL, ES, PL, and JRF designed and produced the small molecule STAT3 inhibitor FLLL32. JF designed and carried out the quantitative real time PCR experiments. WK assisted in experimental design. CL conceived of the study, assisted in experimental design, and helped draft the manuscript. All authors read and approved the final manuscript.

## Pre-publication history

The pre-publication history for this paper can be accessed here:

http://www.biomedcentral.com/1471-2407/11/112/prepub

## Supplementary Material

Additional file 1 **Curcumin and FLLL32 downregulated the expression of STAT3 protein without significant inhibition of STAT3 mRNA transcript levels**. OSA8 cells were treated with 10 μM curcumin or 10 μM FLLL32 and were collected at 4 and 24 hours after treatment, and real-time PCR for STAT3 mRNA was performed. Bars represent STAT3 relative expression (2^-Delta Ct). Experiments were performed in triplicate and repeated three times. The difference between treatment groups and DMSO control group was analyzed using the Students *t *test. *P *values of < 0.05 were considered statistically significant. There was no statistical significance between the treatment groups. Click here for file

Additional file 2 **Treatment with curcumin or FLLL32 did not significantly alter pERK1/2 or total ERK1/2 levels**. Canine (OSA8) or human (SJSA) OSA cell lines were treated with DMSO, 10 μM curcumin, or increasing concentrations of FLLL32 for 24 hours prior to collection. Protein lysates were generated and separated by SDS-PAGE and western blotting for pERK1/2 (Thr202/Tyr204), total ERK1/2, and β-actin was performed. Experiments were repeated two times. Click here for file

## References

[B1] Marina N Gebhardt M Teot L Gorlick R Biology and therapeutic advances for pediatric osteosarcoma Oncologist 2004 9 4 422 441 10.1634/theoncologist.9-4-422 15266096

[B2] Chun R de Lorimier LP Update on the biology and management of canine osteosarcoma Vet Clin North Am Small Anim Pract 2003 33 3 491 516 10.1016/S0195-5616(03)00021-4 12852233

[B3] Wu PK Chen WM Chen CF Lee OK Haung CK Chen TH Primary osteogenic sarcoma with pulmonary metastasis: clinical results and prognostic factors in 91 patients Jpn J Clin Oncol 2009 39 8 514 522 10.1093/jjco/hyp057 19525290

[B4] Withrow SJ Powers BE Straw RC Wilkins RM Comparative aspects of osteosarcoma. Dog versus man Clin Orthop Relat Res 1991 270 159 168 1884536

[B5] Khanna C Wan X Bose S Cassaday R Olomu O Mendoza A Yeung C Gorlick R Hewitt SM Helman LJ The membrane-cytoskeleton linker ezrin is necessary for osteosarcoma metastasis Nat Med 2004 10 2 182 186 10.1038/nm982 14704791

[B6] MacEwen EG Kutzke J Carew J Pastor J Schmidt JA Tsan R Thamm DH Radinsky R c-Met tyrosine kinase receptor expression and function in human and canine osteosarcoma cells Clin Exp Metastasis 2003 20 5 421 430 10.1023/A:1025404603315 14524531

[B7] Paoloni M Davis S Lana S Withrow S Sangiorgi L Picci P Hewitt S Triche T Meltzer P Khanna C Canine tumor cross-species genomics uncovers targets linked to osteosarcoma progression BMC Genomics 2009 10 625 10.1186/1471-2164-10-625 20028558 PMC2803201

[B8] Levine RA Forest T Smith C Tumor suppressor PTEN is mutated in canine osteosarcoma cell lines and tumors Vet Pathol 2002 39 3 372 378 10.1354/vp.39-3-372 12014501

[B9] Mendoza S Konishi T Dernell WS Withrow SJ Miller CW Status of the p53, Rb and MDM2 genes in canine osteosarcoma Anticancer Res 1998 18 6A 4449 4453 9891508

[B10] Johnson AS Couto CG Weghorst CM Mutation of the p53 tumor suppressor gene in spontaneously occurring osteosarcomas of the dog Carcinogenesis 1998 19 1 213 217 10.1093/carcin/19.1.213 9472714

[B11] Wang YC Zheng LH Ma BA Zhou Y Zhang MH Zhang DZ Fan QY Clinical value of signal transducers and activators of transcription 3 (STAT3) gene expression in human osteosarcoma Acta Histochem 2010 in press 20546860 10.1016/j.acthis.2010.03.002

[B12] Catlett-Falcone R Landowski TH Oshiro MM Turkson J Levitzki A Savino R Ciliberto G Moscinski L Fernandez-Luna JL Nunez G Constitutive activation of Stat3 signaling confers resistance to apoptosis in human U266 myeloma cells Immunity 1999 10 1 105 115 10.1016/S1074-7613(00)80011-4 10023775

[B13] Turkson J Jove R STAT proteins: novel molecular targets for cancer drug discovery Oncogene 2000 19 56 6613 6626 10.1038/sj.onc.1204086 11426647

[B14] Fossey SL Liao AT McCleese JK Bear MD Lin J Li PK Kisseberth WC London CA Characterization of STAT3 activation and expression in canine and human osteosarcoma BMC Cancer 2009 9 81 10.1186/1471-2407-9-81 19284568 PMC2666757

[B15] Chen CL Loy A Cen L Chan C Hsieh FC Cheng G Wu B Qualman SJ Kunisada K Yamauchi-Takihara K Signal transducer and activator of transcription 3 is involved in cell growth and survival of human rhabdomyosarcoma and osteosarcoma cells BMC Cancer 2007 7 111 10.1186/1471-2407-7-111 17598902 PMC1964761

[B16] Ryu K Susa M Choy E Yang C Hornicek FJ Mankin HJ Duan Z Oleanane triterpenoid CDDO-Me induces apoptosis in multidrug resistant osteosarcoma cells through inhibition of Stat3 pathway BMC Cancer 2010 10 187 10.1186/1471-2407-10-187 20459702 PMC2874784

[B17] Ryu K Choy E Yang C Susa M Hornicek FJ Mankin H Duan Z Activation of signal transducer and activator of transcription 3 (Stat3) pathway in osteosarcoma cells and overexpression of phosphorylated-Stat3 correlates with poor prognosis J Orthop Res 2010 28 7 971 978 20063378 10.1002/jor.21088

[B18] Gota VS Maru GB Soni TG Gandhi TR Kochar N Agarwal MG Safety and pharmacokinetics of a solid lipid curcumin particle formulation in osteosarcoma patients and healthy volunteers J Agric Food Chem 2010 58 4 2095 2099 10.1021/jf9024807 20092313

[B19] Walters DK Muff R Langsam B Born W Fuchs B Cytotoxic effects of curcumin on osteosarcoma cell lines Invest New Drugs 2008 26 4 289 297 10.1007/s10637-007-9099-7 18071634

[B20] Epstein J Sanderson IR Macdonald TT Curcumin as a therapeutic agent: the evidence from in vitro, animal and human studies Br J Nutr 2010 103 1545 1557 10.1017/S0007114509993667 20100380

[B21] Patel BB Majumdar AP Synergistic role of curcumin with current therapeutics in colorectal cancer: minireview Nutr Cancer 2009 61 6 842 846 10.1080/01635580903285106 20155625 PMC6370343

[B22] Villegas I Sanchez-Fidalgo S Alarcon de la Lastra C New mechanisms and therapeutic potential of curcumin for colorectal cancer Mol Nutr Food Res 2008 52 9 1040 1061 10.1002/mnfr.200700280 18655004

[B23] Chakravarti N Kadara H Yoon DJ Shay JW Myers JN Lotan D Sonenberg N Lotan R Differential Inhibition of Protein Translation Machinery by Curcumin in Normal, Immortalized, and Malignant Oral Epithelial Cells Cancer Prev Res (Phila Pa) 2010 3 331 338 10.1158/1940-6207.CAPR-09-0076 PMC2833226 20145189

[B24] Glienke W Maute L Wicht J Bergmann L Curcumin inhibits constitutive STAT3 phosphorylation in human pancreatic cancer cell lines and downregulation of survivin/BIRC5 gene expression Cancer Invest 2010 28 2 166 171 10.3109/07357900903287006 20121547

[B25] Li QF Zheng YB Yang HB Shi SL Zhao ZL Chen JA [Changes of nuclear matrix proteins during apoptosis of human osteosarcoma MG-63 cells induced by curcumin] Fen Zi Xi Bao Sheng Wu Xue Bao 2008 41 6 473 481 19137819

[B26] Leow PC Tian Q Ong ZY Yang Z Ee PL Antitumor activity of natural compounds, curcumin and PKF118-310, as Wnt/beta-catenin antagonists against human osteosarcoma cells Invest New Drugs 2009 28 766 782 10.1007/s10637-009-9311-z 19730790

[B27] Aggarwal BB Kumar A Bharti AC Anticancer potential of curcumin: preclinical and clinical studies Anticancer Res 2003 23 1A 363 398 12680238

[B28] Kim HY Park EJ Joe EH Jou I Curcumin suppresses Janus kinase-STAT inflammatory signaling through activation of Src homology 2 domain-containing tyrosine phosphatase 2 in brain microglia J Immunol 2003 171 11 6072 6079 14634121 10.4049/jimmunol.171.11.6072

[B29] Lin HJ Su CC Lu HF Yang JS Hsu SC Ip SW Wu JJ Li YC Ho CC Wu CC Curcumin blocks migration and invasion of mouse-rat hybrid retina ganglion cells (N18) through the inhibition of MMP-2, -9, FAK, Rho A and Rock-1 gene expression Oncol Rep 2010 23 3 665 670 10.3892/or_00000679 20127004

[B30] Seo JH Jeong KJ Oh WJ Sul HJ Sohn JS Kim YK Cho do Y Kang JK Park CG Lee HY Lysophosphatidic acid induces STAT3 phosphorylation and ovarian cancer cell motility: their inhibition by curcumin Cancer Lett 2010 288 1 50 56 10.1016/j.canlet.2009.06.023 19647363

[B31] Lin SS Lai KC Hsu SC Yang JS Kuo CL Lin JP Ma YS Wu CC Chung JG Curcumin inhibits the migration and invasion of human A549 lung cancer cells through the inhibition of matrix metalloproteinase-2 and -9 and Vascular Endothelial Growth Factor (VEGF) Cancer Lett 2009 285 2 127 133 10.1016/j.canlet.2009.04.037 19477063

[B32] Yadav VR Suresh S Devi K Yadav S Novel formulation of solid lipid microparticles of curcumin for anti-angiogenic and anti-inflammatory activity for optimization of therapy of inflammatory bowel disease J Pharm Pharmacol 2009 61 3 311 321 10.1211/jpp.61.03.0005 19222903

[B33] Lee DS Lee MK Kim JH Curcumin induces cell cycle arrest and apoptosis in human osteosarcoma (HOS) cells Anticancer Res 2009 29 12 5039 5044 20044614

[B34] Sharma RA Steward WP Gescher AJ Pharmacokinetics and pharmacodynamics of curcumin Adv Exp Med Biol 2007 595 453 470 full_text 17569224 10.1007/978-0-387-46401-5_20

[B35] Fuchs JR Pandit B Bhasin D Etter JP Regan N Abdelhamid D Li C Lin J Li PK Structure-activity relationship studies of curcumin analogues Bioorg Med Chem Lett 2009 19 7 2065 2069 10.1016/j.bmcl.2009.01.104 19249204

[B36] Liang G Shao L Wang Y Zhao C Chu Y Xiao J Zhao Y Li X Yang S Exploration and synthesis of curcumin analogues with improved structural stability both in vitro and in vivo as cytotoxic agents Bioorg Med Chem 2009 17 6 2623 2631 10.1016/j.bmc.2008.10.044 19243951

[B37] Ravindran J Subbaraju GV Ramani MV Sung B Aggarwal BB Bisdemethylcurcumin and structurally related hispolon analogues of curcumin exhibit enhanced prooxidant, anti-proliferative and anti-inflammatory activities in vitro Biochem Pharmacol 2010 79 1658 1666 10.1016/j.bcp.2010.01.033 20138025 PMC2846970

[B38] Lin L Hutzen B Zuo M Ball S Deangelis S Foust E Pandit B Ihnat MA Shenoy SS Kulp S Novel STAT3 phosphorylation inhibitors exhibit potent growth-suppressive activity in pancreatic and breast cancer cells Cancer Res 2010 70 6 2445 2454 10.1158/0008-5472.CAN-09-2468 20215512 PMC2843552

[B39] Lin L Deangelis S Foust E Fuchs J Li C Li PK Schwartz EB Lesinski GB Benson D Lu J A novel small molecule inhibits STAT3 phosphorylation and DNA binding activity and exhibits potent growth suppressive activity in human cancer cells Mol Cancer 2010 9 217 10.1186/1476-4598-9-217 20712901 PMC2936338

[B40] Bill MA Fuchs JR Li C Yui J Bakan C Benson DM Jr Schwartz EB Abdelhamid D Lin J Hoyt DG The small molecule curcumin analog FLLL32 induces apoptosis in melanoma cells via STAT3 inhibition and retains the cellular response to cytokines with anti-tumor activity Mol Cancer 2010 9 165 10.1186/1476-4598-9-165 20576164 PMC2902420

[B41] Liu Y Fuchs J Li C Lin J IL-6, a risk factor for hepatocellular carcinoma: FLLL32 inhibits IL-6-induced STAT3 phosphorylation in human hepatocellular cancer cells Cell Cycle 2010 9 17 3423 3427 10.4161/cc.9.17.12946 20818158

[B42] Wei CC Ball S Lin L Liu A Fuchs JR Li PK Li C Lin J Two small molecule compounds, LLL12 and FLLL32, exhibit potent inhibitory activity on STAT3 in human rhabdomyosarcoma cells Int J Oncol 2011 38 1 279 285 21109950

[B43] Modiano JF Breen M Lana SE Ehrhart N Fosmire SP Thomas R Jubala CM Lamerato-Kozicki AR Ehrhart EJ Schaack J Duke RC Cutter GC Bellgrau D Naturally occurring translational models for development of cancer gene therapy Gene Ther Mol Biol 2006 10 31 40

[B44] Thomas R Scott A Langford CF Fosmire SP Jubala CM Lorentzen TD Hitte C Karlsson EK Kirkness E Ostrander EA Construction of a 2-Mb resolution BAC microarray for CGH analysis of canine tumors Genome Res 2005 15 12 1831 1837 10.1101/gr.3825705 16339382 PMC1356122

[B45] Livak KJ Schmittgen TD Analysis of relative gene expression data using real-time quantitative PCR and the 2(-Delta Delta C(T)) Method Methods 2001 25 4 402 408 10.1006/meth.2001.1262 11846609

[B46] Heitz F Harter P Ewald-Riegler N Papsdorf M Kommoss S du Bois A Poly(ADP-ribosyl)ation polymerases: mechanism and new target of anticancer therapy Expert Rev Anticancer Ther 2010 10 7 1125 1136 10.1586/era.10.53 20645701

[B47] Saba NS Levy LS Apoptotic induction in B-cell acute lymphoblastic leukemia cell lines treated with a protein kinase C inhibito Leuk Lymphoma 2011 Jan 27 [Epub ahead of print] 21271861 10.3109/10428194.2011.552136

[B48] Ichiba M Nakajima K Yamanaka Y Kiuchi N Hirano T Autoregulation of the Stat3 gene through cooperation with a cAMP-responsive element-binding protein J Biol Chem 1998 273 11 6132 6138 10.1074/jbc.273.11.6132 9497331

[B49] Ulane CM Rodriguez JJ Parisien JP Horvath CM STAT3 ubiquitylation and degradation by mumps virus suppress cytokine and oncogene signaling J Virol 2003 77 11 6385 6393 10.1128/JVI.77.11.6385-6393.2003 12743296 PMC155014

[B50] Safhi MM Rutherford C Ledent C Sands WA Palmer TM Priming of Signal Transducer and Activator of Transcription Proteins for Cytokine-Triggered Polyubiquitylation and Degradation by the A2a Adenosine Receptor Mol Pharmacol 2010 77 968 978 10.1124/mol.109.062455 20185553

[B51] Milacic V Banerjee S Landis-Piwowar KR Sarkar FH Majumdar AP Dou QP Curcumin inhibits the proteasome activity in human colon cancer cells in vitro and in vivo Cancer Res 2008 68 18 7283 7292 10.1158/0008-5472.CAN-07-6246 18794115 PMC2556983

[B52] Darnowski JW Goulette FA Guan YJ Chatterjee D Yang ZF Cousens LP Chin YE Stat3 cleavage by caspases: impact on full-length Stat3 expression, fragment formation, and transcriptional activity J Biol Chem 2006 281 26 17707 17717 10.1074/jbc.M600088200 16636048

[B53] Chakravarti N Myers JN Aggarwal BB Targeting constitutive and interleukin-6-inducible signal transducers and activators of transcription 3 pathway in head and neck squamous cell carcinoma cells by curcumin (diferuloylmethane) Int J Cancer 2006 119 6 1268 1275 10.1002/ijc.21967 16642480

[B54] Blasius R Reuter S Henry E Dicato M Diederich M Curcumin regulates signal transducer and activator of transcription (STAT) expression in K562 cells Biochem Pharmacol 2006 72 11 1547 1554 10.1016/j.bcp.2006.07.029 16959222

[B55] Maiti K Mukherjee K Gantait A Saha BP Mukherjee PK Curcumin-phospholipid complex: Preparation, therapeutic evaluation and pharmacokinetic study in rats Int J Pharm 2007 330 1-2 155 163 10.1016/j.ijpharm.2006.09.025 17112692

[B56] Liu AC Zhao LX Zhai GX Lou HX Du JS [An investigation on formation mechanisms and preparation of curcumin phospholipid complex] Zhongguo Zhong Yao Za Zhi 2008 33 17 2112 2117 19066053

[B57] Lin L Hutzen B Ball S Foust E Sobo M Deangelis S Pandit B Friedman L Li C Li PK New curcumin analogues exhibit enhanced growth-suppressive activity and inhibit AKT and signal transducer and activator of transcription 3 phosphorylation in breast and prostate cancer cells Cancer Sci 2009 100 9 1719 1727 10.1111/j.1349-7006.2009.01220.x 19558577 PMC11158315

[B58] Friedman L Lin L Ball S Bekaii-Saab T Fuchs J Li PK Li C Lin J Curcumin analogues exhibit enhanced growth suppressive activity in human pancreatic cancer cells Anticancer Drugs 2009 20 6 444 449 10.1097/CAD.0b013e32832afc04 19384191 PMC2855307

[B59] Cen L Hutzen B Ball S DeAngelis S Chen CL Fuchs JR Li C Li PK Lin J New structural analogues of curcumin exhibit potent growth suppressive activity in human colorectal carcinoma cells BMC Cancer 2009 9 99 10.1186/1471-2407-9-99 19331692 PMC2674881

[B60] Zhao ZL Li QF Zheng YB Chen LY Shi SL Jing GJ The aberrant expressions of nuclear matrix proteins during the apoptosis of human osteosarcoma cells Anat Rec (Hoboken) 2010 293 5 813 820 20340094 10.1002/ar.21074

[B61] Xie TX Huang FJ Aldape KD Kang SH Liu M Gershenwald JE Xie K Sawaya R Huang S Activation of stat3 in human melanoma promotes brain metastasis Cancer Res 2006 66 6 3188 3196 10.1158/0008-5472.CAN-05-2674 16540670

[B62] Oda Y Yamamoto H Tamiya S Matsuda S Tanaka K Yokoyama R Iwamoto Y Tsuneyoshi M CXCR4 and VEGF expression in the primary site and the metastatic site of human osteosarcoma: analysis within a group of patients, all of whom developed lung metastasis Mod Pathol 2006 19 5 738 745 10.1038/modpathol.3800587 16528367

[B63] Kaya M Wada T Akatsuka T Kawaguchi S Nagoya S Shindoh M Higashino F Mezawa F Okada F Ishii S Vascular endothelial growth factor expression in untreated osteosarcoma is predictive of pulmonary metastasis and poor prognosis Clin Cancer Res 2000 6 2 572 577 10690541

