# Supplementary figures and images for: The novel curcumin analog FLLL32 decreases STAT3 DNA binding activity and expression, and induces apoptosis in osteosarcoma cell lines
Source: BMC Cancer. 2011 Mar 28;11:112. doi: 10.1186/1471-2407-11-112 (PMC3074561; doi:10.1186/1471-2407-11-112)

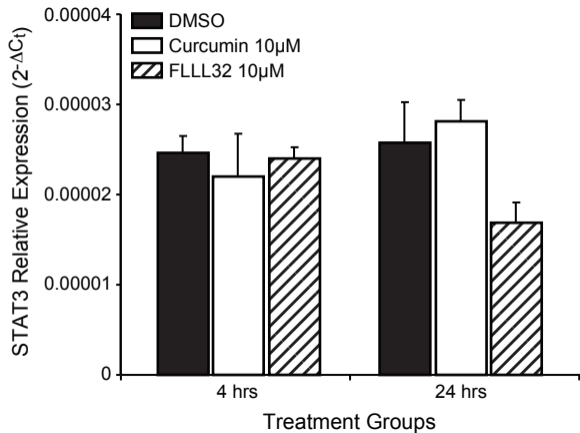

Supplement: Additional file 1 — Curcumin and FLLL32 downregulated the expression of STAT3 protein without significant inhibition of STAT3 mRNA transcript levels. OSA8 cells were treated with 10 μM curcumin or 10 μM FLLL32 and were collected at 4 and 24 hours after treatment, and real-time PCR for STAT3 mRNA was performed. Bars represent STAT3 relative expression (2^-Delta Ct). Experiments were performed in triplicate and repeated three times. The difference between treatment groups and DMSO control group was analyzed using the Students t test. P values of < 0.05 were considered statistically significant. There was no statistical significance between the treatment groups. [file 1471-2407-11-112-S1.PDF]

## OSA8

Curcumin ( $\mu\text{M}$ )

-      10      -      -      -

FLLL32 ( $\mu\text{M}$ )

-      -      2.5      5      10

pERK1/2

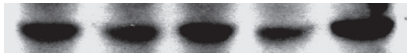

ERK1/2

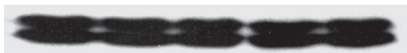

$\beta$ -actin

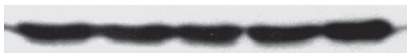

## SJSA

pERK1/2

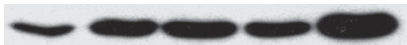

ERK1/2

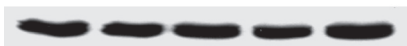

$\beta$ -actin

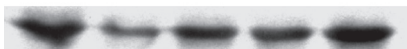

Supplement: Additional file 2 — Treatment with curcumin or FLLL32 did not significantly alter pERK1/2 or total ERK1/2 levels. Canine (OSA8) or human (SJSA) OSA cell lines were treated with DMSO, 10 μM curcumin, or increasing concentrations of FLLL32 for 24 hours prior to collection. Protein lysates were generated and separated by SDS-PAGE and western blotting for pERK1/2 (Thr202/Tyr204), total ERK1/2, and β-actin was performed. Experiments were repeated two times. [file 1471-2407-11-112-S2.PDF]
